# A Design of Experiment Approach for Surface Roughness Comparisons of Foam Injection-Moulding Methods

**DOI:** 10.3390/ma13102358

**Published:** 2020-05-20

**Authors:** Gethin Llewelyn, Andrew Rees, Christian Griffiths, Martin Jacobi

**Affiliations:** 1 College of Engineering, Swansea University, Swansea, Wales SA1 8EN, UK; Andrew.Rees@Swansea.ac.uk (A.R.); c.a.griffiths@swansea.ac.uk (C.G.); 2 Trexel GmbH, Ahlefelderstr. 64, D-51645 Gummersbach, Germany; m.jacobi@trexel.com

**Keywords:** polypropylene, talc, TecoCell^®^, MuCell^®^, foam Injection Moulding

## Abstract

The pursuit of polymer parts produced through foam injection moulding (FIM) that have a comparable surface roughness to conventionally processed components are of major relevance to expand the application of FIM. Within this study, 22% talc-filled copolymer polypropylene (PP) parts were produced through FIM using both a physical and chemical blowing agent. A design of experiments (DoE) was performed whereby the processing parameters of mould temperatures, injection speeds, back-pressure, melt temperature and holding time were varied to determine their effect on surface roughness, Young’s modulus and tensile strength. The results showed that mechanical performance can be improved when processing with higher mould temperatures and longer holding times. Also, it was observed that when utilising chemical foaming agents (CBA) at low-pressure, surface roughness comparable to that obtained from conventionally processed components can be achieved. This research demonstrates the potential of FIM to expand to applications whereby weight saving can be achieved without introducing surface defects, which has previously been witnessed within FIM.

## 1. Introduction

Recent demands from lowering polymer consumption and making lightweight parts has seen the rise of foam injection moulding (FIM) through different foaming techniques [1,2,3]. FIM can be performed through either physical blowing agents (PBA) or chemical blowing agents (CBA) [4]. PBA is used by injecting a super critical gas through the moulding barrel while the polymer is being metered, in order to form a single-phase solution [5]; whilst CBA are added to the parent material in small amounts prior to processing [6]. The introduction of FIM does result in component weight saving, however it also lowers the mechanical properties and introduces the surface defect of swirl marks [7]. The swirl marks can be attributed to the mould being filled by the polymer/gas solution. In particular, cell nucleation has been initiated at this stage due to the rapid pressure drop at the injection point. Following this, the fountain flow affect freezes and stretches the cells at the mould/polymer interface resulting in swirl marks [8]. 

Traditionally, FIM removes the packing phase which is witnessed in conventional injection moulding (IM). This is removed as essentially the packing phase is completed by the foaming of the polymer. This method is referred to as low-pressure FIM and it was the original technique used when the technology was first developed [9]. The technique has introduced weight savings of up to 15% within thermoplastics such as polypropylene (PP) [10]. At present, this technique is the most utilised in an industrial capacity, however recent developments in alternative techniques have seen the position challenged. 

Recent developments in FIM have seen the introduction of high-pressure processing. During high-pressure processing, the packing phase is not removed from the conventional IM process, but instead reduced or kept constant. Like low-pressure FIM, the single-phase solution begins to nucleate during the filling stage of the injection cycle through pressure changes. However, the introduction of the packing stage causes the pre-nucleated cells to re-dissolve back into the melt. This occurs if the packing pressure is kept above the solubility pressure of the PBA, the pre-nucleated cells can be re-dissolved into the polymer and nucleated in situ within the mould [11]. Finally, the holding pressure is stopped, and the cells nucleate through either thermal shrinkage through normal cooling or by a secondary pressure drop; usually by mould opening [12]. Although the resulting part weight reductions cannot meet the level of low-pressure FIM, the technique has been shown to improve the cellular properties [11,12].

The use of semi-crystalline polymers, such as PP, are extensively used within the plastics industry due to their high thermal stability, moisture resistance, excellent chemical and corrosion resistance, ease of processability and low density [13]. However, with the additional functionality requirements in recent years, it is becoming more difficult to use PP in its neat state. Instead, fillers are added to increase structural properties [14]. Moreover, unfilled PP experiences poor foaming behaviour due to it being a linear hydro-carbon polymer which has poor melt strength and low viscoelastic properties, leading to cell coalescence and resulting in poor cellular structures [15,16,17]. Therefore, fillers such as silica, carbon black, calcium carbonate and talc have all been included in the FIM of polymers with poor foaming properties to improve the isothermal crystallisation process which improves the nucleation and resulting cellular structures [18,19,20]. 

Within the current knowledge base, limited work has been performed on the effect of high-pressure FIM on the resulting mechanical properties and the surface roughness of the final part. Shaayegan et al. investigated high-pressure foaming, whereby cellular properties were dramatically increased if the mould opening is utilised to initiate a secondary pressure drop [21]. Further research has demonstrated that with an increase in packing time from 4 s to 8 s in FIM, polystyrene (PS) with CO_2_ as the blowing agent caused the average cell size to drop and cell density to increase [22]. In addition, it has been proven that through high pressure FIM, nano cellular structures are obtainable [23,24,25]. Costeux has highlighted the levels at which nanofoams have been produced in recent years within the foam industry [26]. In a further study, Ameli et al. achieved a minimum cell size of 70 nm and maximum cell density of 2 × 10^14^ with a PP/Multi-Walled Carbon Nanotubes mixture [27]. However, most of these studies are through laboratory-based procedures, such as batch foaming, and have yet to be achieved through industrial foaming routes, like injection moulding. 

Obtaining FIM parts that have comparable surface roughness as their conventionally processed solid counterpart has been investigated. In particular, various methods to improve these surface finish defects have been attempted through co-injection moulding [28], gas counter pressure [29] and vario-thermal moulding [30,31]. All process variants have shown to improve the FIM parts surface appearance. However, the improvement in surface finish has introduced detrimental effects with regards to cycle time and environmental aspects. Therefore, these technologies have seen limited impact within an industrial context and have led researchers to continue the pursuit of improvements in surface finish when processing through FIM. 

Lee et al. have achieved near perfect surface finish polyethylene (PE) parts with FIM. This was achieved by optimising processing whereby 0.173 wt.% of supercritical N_2_ is applied [32]. More recently, Guo et al., proposed that through in-mould decoration, foamed PP could be produced that had the same surface appearance as solid parts [33]. In another study, Wang et al. used a multi stage process to create a defect-free surface with a nanocellular microstructure through incorporating polytetrafluoroethylene (PTFE) into the PP matrix through in situ nanofibrillation [23]. Although research in FIM has advanced greatly in recent years, there is yet to be a study that has demonstrated that FIM can achieve surface roughness comparable to conventional IM when moulding components in PP with high weight savings.

In this research, a 22% talc-filled PP was processed initially through conventional injection moulding (IM) then produced using variations of FIM process. For the PBA, super critical N_2_ was used (through the MuCell^®^ system) and the CBA used was TecoCell^®^ H1. In addition, for the FIM processing a design of experiment (DoE) varying the 5 main processing parameters of mould temperature, injection speed, back-pressure, melt temperature and holding time were performed to investigate the effect of varying processing parameters on the resulting tensile strength and surface roughness. 

## 2. Materials and Methods 

### 2.1. Materials

**Figure 1:**
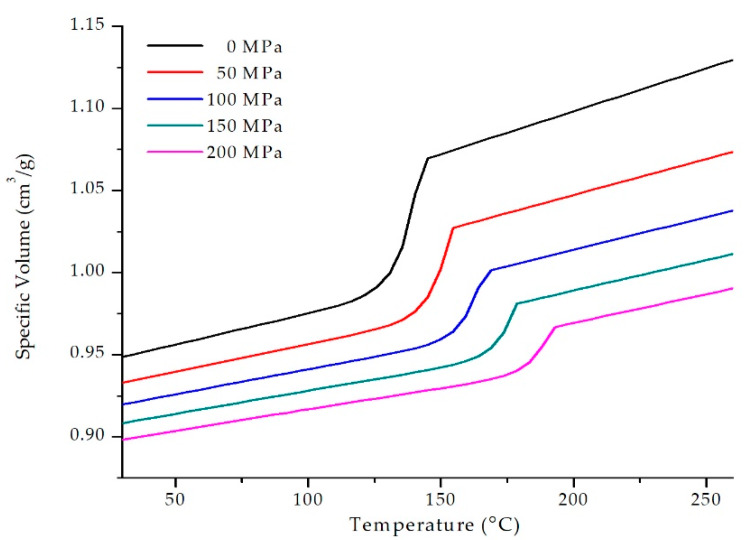
Pressure-Volume-Temperature data of the copolymer polypropylene used in this research.

This research has used a commercial grade copolymer polypropylene (PP), synthesised with 22% talc and black masterbatch as the base material. It has a melt flow rate (MFR) of 35 g/10 min and a density (ρ) of 1.05 g/cm^3^. The pressure-volume-temperature (PvT) data of this polymer can be seen in Figure 1. 

For the CBA experiments, an endothermic CBA was used: TecoCell^®^ H1 (Trexel GmbH, Gummersbach, Germany), which was added to the base material. This CBA creates an endothermic reaction when heated within the injection moulding barrel above 200 °C; between monosodium citrate (C_6_H_7_NaO_7_) and calcium carbonate (CaCO_3_) [6]; releasing carbon dioxide (CO_2_) into the IM barrel.

**Figure 2:**
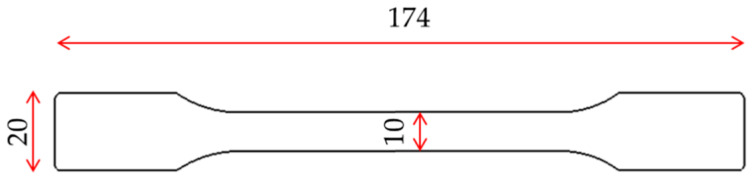
Moulded part geometry, thickness is 4 (dimensions in millimetres).

For the PBA experiments, a gas dosing unit (T100, Trexel GmbH, Gummersbach, Germany) was used with nitrogen (N_2_) of 99.998% purity, to produce MuCell^®^ injection moulded parts. For all of the experiments performed in this research, the polymer was dried for 4 h at 80 °C prior to processing. In each moulding cycle, 2 tensile bars complying to type A1 within BS EN ISO 2073:2014 were produced (Figure 2) and mould layout can be seen in the authors’ previous publication [19]. The Injection Moulding machine used for this research was a 40 mm screw diameter IM machine (e-Victory 120, ENGEL, Warwick, UK); with a maximum clamping force of 1200 kN.

### 2.2. Experimental Procedure

To evaluate the resulting tensile strength and surface roughness when using a PBA, a full 2^5^ factorial DoE was used by varying the mould temperatures, injection speeds, barrel back-pressure, melt temperature and injection packing time (factorial design Table A1, maximum and minimum values displayed in Table 1). 

Similar to other research, preliminary experiments were performed to obtain the maximum and minimum input values when utilising a PBA [34]. The ‘1’ values shown Table 1 for mould temperature and injection speeds are limited to the equipment available, while the other maximum values are limited by the polymer. The ‘−1’ values for all the parameters were limited by the polymer; recommended by the material supplier. Also, conventional injection moulded parts were moulded as an experimental reference in terms of the weight savings of the foamed parts. The target weight savings of the final parts in relation to the conventional IM, were 12.6% and 8.8% for the 0 s and 7 s holding time, respectively. For all experiments the clamping force was kept consistent at 1000 kN. In addition, for the PBA experiments 40 mg of supercritical N_2_ was used. All of the experiment settings were repeated 25 times to ensure the process was repeatable; of which 5 of these samples were tested at random all testing procedures used in this research, 

Following the characterisation of the optimum processing condition for minimum achievable surface roughness when utilising a PBA, the same parameters were used for the CBA experiments. 

### 2.3. Characterisation Methods

#### 2.3.1. Tensile Properties

Tensile tests were performed on a mechanical testing unit (H25 KS, Hounsfield, Surrey, UK) with BS EN ISO 527–1:2012 compliance, to obtain the maximum stress and subsequently to calculate the Young’s modulus (E). The ultimate tensile stress (S_u_) was obtained by dividing the maximum force value by cross-sectional area. While the Young’s modulus (E) was obtained by taking the strain values between 0.0005 and 0.0025, collected using an axial extensometer (3542, Epsilon, WY, USA), and using the chord slope method of the stress obtained from the force values within this range. This was run using 1 mm/min until a strain of 0.0025 had been met, then increased to 10 mm/min until fracture. 

#### 2.3.2. Part Surface Roughness

The resulting surface characteristics were quantified through surface roughness measurements. This was completed using a surface profilometer (Dektak 150, Veeco UK, St. Ives, UK) using 4 mg of force along 10 mm (L) with the resulting height being y. The calculated surface roughnesses were the arithmetical average deviation from the mean line (R_a_), and the root mean squared value of the roughness (R_q_); seen below in Equations (1) and (2), respectively.

(1) $$R_{a}=\frac{1}{L}\overset{x=L}{\underset{x=0}{\int }}\left|y\right|\;\mathrm{dx}$$

(2) $$R_{q}=\sqrt{\overset{x=L}{\underset{x=0}{\int }}y^{2}\left(x\right)\;\mathrm{dx}}$$

## 3. Results and Discussion

Initially within this section the main findings from the DoE activities for the processing with a PBA are presented. Then, the results from the two CBAs are compared to the best surface finish appearance PBA parts and the conventionally moulded parts. The DoE data is presented as simplified regression models using the analysis of variance (ANOVA) model. This is a method to identify which of the processing parameters have the highest statistical influence on the process. The values are generated using the experimental values in a sum of squares and divided by the numbers of degrees of freedom of each error: the variance for each parameter is then compared with the variance of the error [35]. This DoE data is also shown in interaction plots; whereby the input processing parameters are compared against each other to determine whether there is an interaction between processing parameters which alter the final results [36]. Finally, the regression models derived from the DoE data can be seen in Table A2 in Appendix A. 

### 3.1. Tensile Strength

#### 3.1.1. Modulus of Elasticity (E)

**Figure 3:**
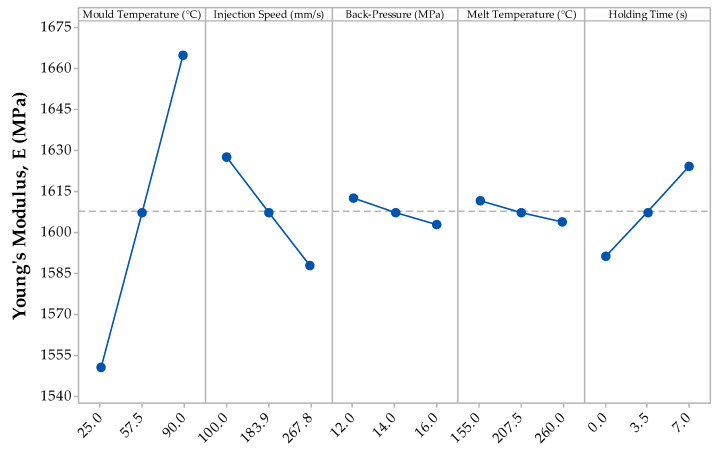
Main effects plot for the mean Young’s modulus (E) results from the DoE.

Figure 3 displays the resulting mean Young’s modulus (E) against the five variable processing parameters from the DoE: mould temperature, injection speeds, back-pressure, melt temperature and holding time.

The DoE results for the Young’s modulus clearly identify the effect of each of the five input processing parameters. In particular, with increasing mould temperature and holding time, the Young’s modulus of the part increases. However, with an increase in the injection speed, back-pressure and melt temperature, the Young’s modulus reduces. The most significant input processing parameter on the resulting Young’s modulus is that of mould temperature, a range of 116.7 MPa while the least significant input processing parameter range is 7.6 MPa from the melt temperature. The information within Table 2 confirms the viability of this data with a P-value below 0.05; showing that the confidence level of this parameter having an effect on the outcome being highly significant [37].

Within this research, all tensile strength factors and interactions with a P-value lower than that of the confidence level (α = 0.05) are deemed significant. Therefore, they are seen to have a great effect on the response when the testing level is moved from low to high or vice versa [38]. 

The data in Table 2 display the significant data from the analysis of variance model which shows all of the input processing parameters, along with any other interaction between these parameters which show a P-value lower than 0.05. The data validates the analysis from Figure 1 that the mould temperature is the most significant variable as it has a very low P-value of less than 0.001. The only significant factors were that of 2-way interactions; with 3- to 5-way interactions having very high P values and hence, not being of any significance. Holding time and injection speed were the other 2 variables that showed significant effects with *p*-value of 0.003 and 0.011, respectively. Finally, as shown in both Table 2 and Figure 3, the least significant variables were those of back-pressure and melt temperature with *p*-values of 0.893 and 0.331, respectively. As for the 2-way interaction plots, the most significant was that of melt temperature with holding time, followed closely by that of injection speed with melt temperature. Injection speed combined with holding time also showed significance effects, along with back-pressure combined with melt temperature.

It is plausible that mould temperature having such a large effect on the Young’s modulus can be attributed to the presence of shish-kebab microstructures typically witnessed at higher cooling rate in the sample interior as it inhibits the relaxation of the crystalline structure prior to crystallisation [39].

#### 3.1.2. Ultimate Tensile Strength (S_u_)

**Figure 4:**
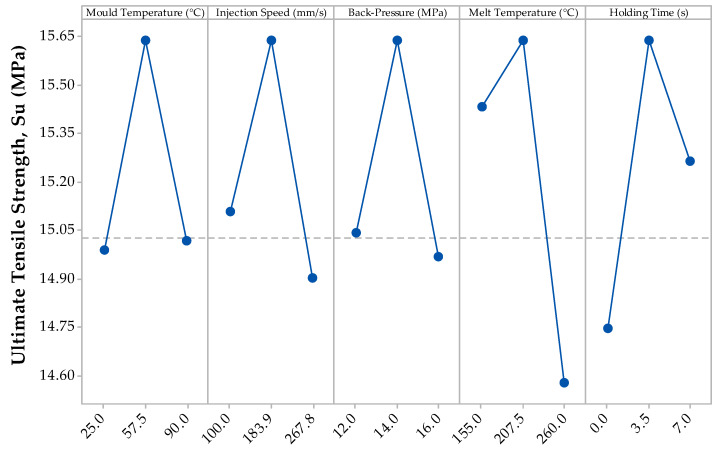
Main effects plot for the mean maximum tensile strength (S_u_) results from the DoE.

Figure 4 shows the results of the ultimate tensile strength (S_u_) from the DoE of the PBA. 

Unlike Figure 3, the mean S_u_ data shows a weaker trend, compared to the Young’s Modulus data, due to the input processing parameters due to the midpoint DoE processing setting being 15.64 MPa; a higher mean value than all the processing parameters. However, the increase and decrease trends are in line with Young’s modulus data. In particular, mould temperature and holding time increase the mean S_u_ whilst injection speed, back-pressure and melt temperature reduce S_u_. However, the input processing parameter setting with the greatest effect on the S_u_, are that of the melt temperature with 0.85 MPa. Table 3 confirms the observation as the P-value of this processing parameter is close to 0: showing a great significance on the S_u_. 

It has been previously reported that excessive melt temperatures of the single-phase solution causes excessive growth of cells, leading to poor cell microstructure and hence, a lower mechanical strength [40]. This is evident with this research and highlights the importance of cooling rates with FIM for tensile strength.

### 3.2. Part Surface Roughness

**Figure 5:**
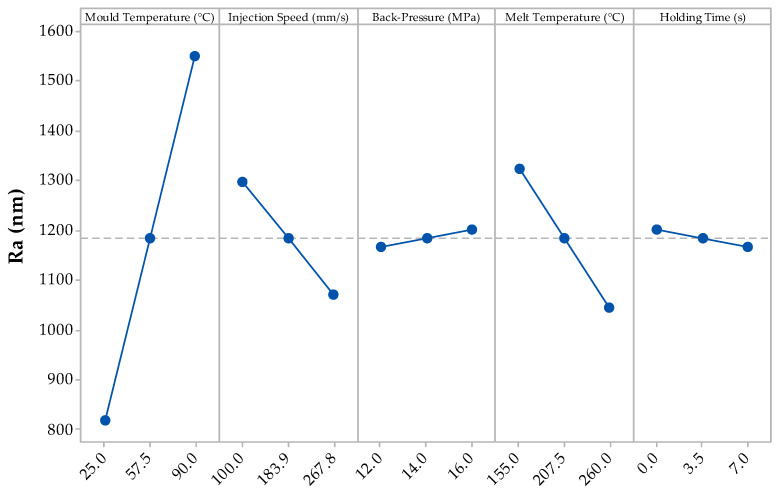
Main effects plot for the R_a_ results from the DoE.

The main effects plot for the mean R_a_ values from the DoE can be seen in Figure 5. 

As the mould temperature increases from 25 °C to 90 °C, the mean R_a_ also increases from 0.819 µm to 1.550 µm. This result can be explained whereby the higher mould temperatures delay the formation of the frozen front; hence the polymer/gas solution continues to nucleate towards the moulding surface causing a pitted surface which causes an increase in surface roughness. A further explanation is that this is post-blow: whereby the gas diffuses out of the part after the moulding process as the polymer continues to balance its thermodynamic boundaries [41,42]. Furthermore, when injection speed is increased from 100 mm/s to 267.8 mm/s, the mean R_a_ decreased from 1.297 µm to 1.072 µm. This reduction in surface roughness with an increase in injection speed can be attributed to the cavity being filled earlier hence the frozen layer may have started to freeze thus mitigating the polymer/gas solution from reaching the moulding surface [43,44]. When processing with a higher melt temperature, the R_a_ decreased from 1.324 µm to 1.045 µm. The processing parameters with the least effect on the mean R_a_ are the back-pressure and the holding time, whereby a contribution of 0.035 µm and 0.032 µm respectively were witnessed on the resulting moulding components. Table 4 and Table 5 show the analysis of variance model results for the R_a_ and R_q_ respectively. 

Again, the *p*-values for each source are similar on both tables; with the mould temperature having the most significance with 0.039 and 0.028, respectively; the only processing parameters that have a major significance (<0.05) on the part surface. The melt temperature and holding time *p*-values demonstrate that they have a negligible effect on the resulting surface roughness. Furthermore, from the results obtained from the linear model, none of the 2- to 5-way interactions have any significance on the R_a_ and R_q_ values. However, when considering the significance of a 2-way interaction, injection speed and back-pressure had *p*-values of 0.1 and 0.088 respectively.

**Figure 6:**
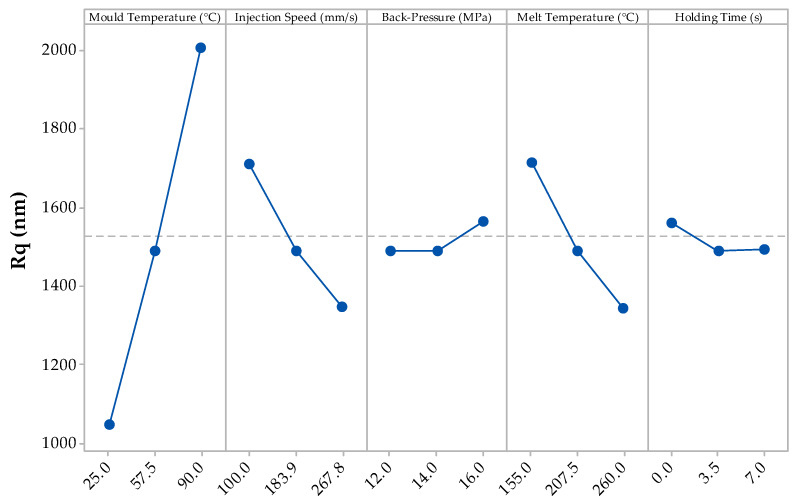
Main effects plot for the R_q_ results from the DoE.

The root mean squared height deviation (R_q_) of the DoE for PBA is presented in Figure 6, whereby the data corresponds to the results presented in Figure 5 except for the mid-point DoE setting not lying on the trend line. However, all the processing parameters have the same effect, with the mould temperature having the greatest effect on the R_q_ and the back-pressure and holding time having negligible effect. 

The R_a_ and R_q_ values of the conventionally moulded part were 0.367 µm and 0.439 µm, respectively. As seen in Figure 5 and Figure 6, many of the mean results from the DoE when processing with FIM yield higher surface roughness profiles. The fast injection speeds typically applied in FIM aided in reducing surface roughness as the resulting filling time is reduced and, therefore, allows the cells to nucleate earlier during the filling stage [10]. The mould temperature had a large effect on the surface profiles as this alters the crystallisation of the polymer/gas solution. With a higher mould temperature, this increases the cooling rate (due to further time required in order to meet the crystallisation temperature), and hence cause different cellular microstructures [32].

### 3.3. Comparison of Physical Blowing Agents to Chemical Blowing Agents

**Figure 7:**
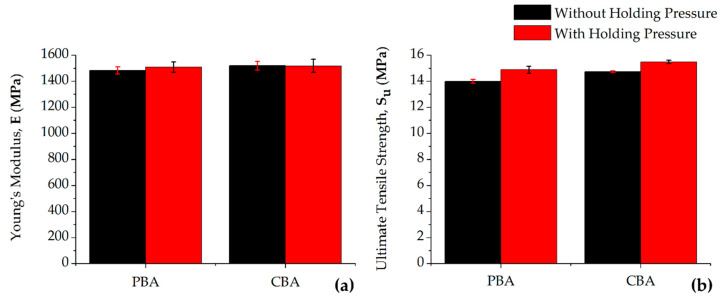
(**a**) Young’s modulus (E) and (**b**) ultimate tensile strength (S_u_) data of a physical blowing agent (PBA) compared to a similarly processed chemical blowing agent (CBA).

Previous work performed by Llewelyn et al. showed that with unfilled and talc-filled PP, superior mechanical properties were witnessed in parts processed with CBA [19]. This trend continues, as shown Figure 7. 

For the CBAs, the processing parameters that resulted in the lowest R_a_ and R_q_ were then used to create FIM parts through a CBA using no holding pressure and holding pressure with 1 wt.%. The main reason why the chemically foamed parts result in higher E and S_u_ values, are due to the thicker skin wall and can be correlated to the modelling theory derived by Xu and Kishbaugh where a thicker skin results in a stronger tensile and flexural part [45]. The physically foamed parts without/with holding pressure in Figure 7 have a skin thickness (T_w_) of 549 µm and 394 µm, respectively. The thinnest T_w_ exhibited in the chemically foamed parts is 775 µm while the thickest is 1060 µm. 

**Figure 8:**
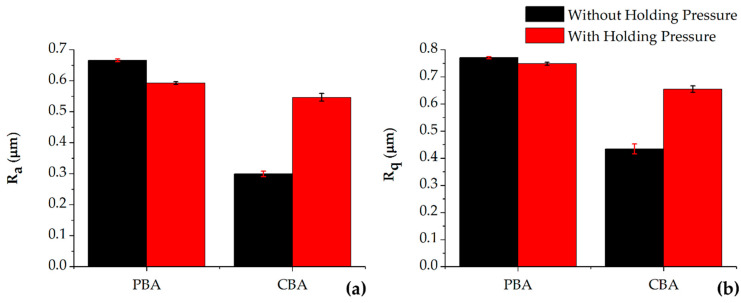
(**a**) R_a_ and (**b**) R_q_ data of a physical blowing agent compared to a similarly processed chemical blowing agent.

The mean R_a_ and R_q_ values for the chemically foamed parts are shown in Figure 8. From the results it can be concluded that when processing with a CBA the addition of holding pressure does not lead to any improvement in surface roughness. Also, the results demonstrate that with the addition of CBA when processed with low pressure, surface roughness can be achieved that ae directly comparable to conventionally moulded parts. 

**Figure 9:**
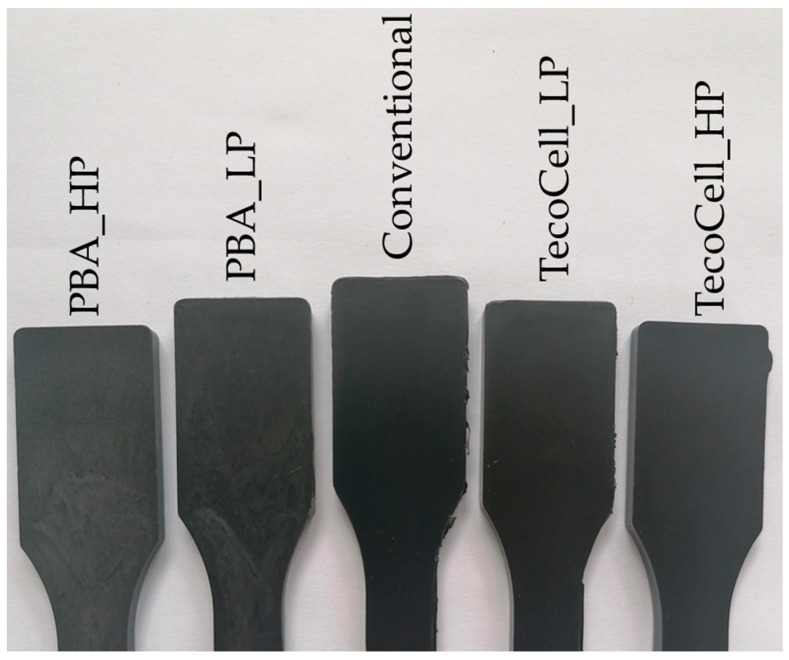
Visual comparison between the conventional, PBA and CBA parts.

Figure 9 shows a visual representation of the parts compared in this section of the research. The parts foamed through the PBA are distinct as they have a much greyer appearance than the other parts; a regular problem exhibited through the PBA. Finally, the parts foamed using the CBA can be seen to have a similar appearance to conventionally moulded part. This is confirmed by the surface roughness measurements as the conventionally moulded parts have an average R_a_ and R_q_ of 0.367 µm and 0.439 µm, while the CBA parts (low-pressure foaming), have 0.299 µm and 0.434 µm, respectively. 

## 4. Conclusions

This work took a talc-filled PP and used low gas dosing in a microcellular injection moulding setup. The initial research performed was a design of experiments with 5 varying input processing parameters: mould temperature, injection speed, back-pressure, melt temperature and holding time. The physically foamed parts with the best surface finishes from this research were compared to similarly manufactured parts through a chemical foaming agent. The major findings from this research are:

- The Young’s modulus is heavily affected by mould temperature, whilst melt temperature has the lowest statistical effect on the process.
- The resulting surface roughness is affected by all 5 of the input processing parameters. The mould temperature was found to have the greatest influence. This can be attributed to the high mould temperatures causing post-blow (gas diffusing out of the part after moulding) [41].
- The parts produced through chemical blowing agents were superior to the physically foamed parts with regards to both mechanical properties and the surface roughness. However, using holding pressure with the chemical foaming resulted in poorer mechanical and surface properties.
- Finally, the parts produced through the chemical foaming agent had resulting surface roughnesses that are comparable to conventionally processed components. This demonstrates the great potential for this technology to be applied in engineering applications where lightweight components are required, as surface roughness will not be compromised.

## Figures and Tables

**Table 1:** 2^5^ Full factorial design of experiment (DoE).

Input Variable	−1	0	1
Mould Temperature (°C)	25	57.5	90
Injection Speed (mm/s)	100	183.9	267.8
Back-Pressure (MPa)	12	14	16
Average Melt Temperature (°C)	155	207.5	260
Holding Time (s)	0	3.5	7

**Table 2:** Analysis of variance model for Young’s modulus against processing parameters.

Source	*df*	Sum of Squares	Mean Squares	F-Value	*p*-Value
*Model*	9	187,000	20,778	14.18	0.000
*Linear*	5	138,934	27,787	18.97	0.000
Mould Temperature	1	114,005	114,005	77.83	0.000
Injection Speed	1	11,190	11,190	7.64	0.011
Back-Pressure	1	27	27	0.02	0.893
Melt Temperature	1	1444	1444	0.99	0.331
Holding Time	1	12,268	12,268	8.37	0.008
*2-Way Interactions*	4	48,066	12,016	8.2	0.000
Injection Speed × Melt Temperature	1	12,591	12,591	8.6	0.007
Injection Speed × Holding Time	1	11,461	11,461	7.82	0.010
Back-Pressure × Melt Temperature	1	7558	7558	5.16	0.033
Melt Temperature × Holding Time	1	16,456	16,456	11.23	0.003
*Residual*	23	33,692	1465		
*Curvature*	1	0	0	0.00	0.994
*Lack-of-Fit*	22	33,692	1531		
*Total*	32	220,692			

**Table 3:** Analysis of variance linear model for ultimate tensile strength against processing parameters.

Source	*df*	Sum of Squares	Mean Squares	F-Value	*p*-Value
*Model*	7	9.5998	1.3714	31.07	0.000
*Linear*	5	8.4735	1.69469	38.39	0.000
Mould Temperature	1	0.0107	0.01068	0.24	0.627
Injection Speed	1	0.3843	0.38433	8.71	0.007
Back-Pressure	1	0.0369	0.03691	0.84	0.369
Melt Temperature	1	5.9321	5.9321	134.38	0.000
Holding Time	1	2.1094	2.10944	47.78	0.000
*2-way interactions*	1	0.7347	0.73466	16.64	0.000
Melt Temperature × Holding Time	1	0.7347	0.73466	16.64	0.000
*Curvature*	1	0.3917	0.39165	8.87	0.006
*Residual*	25	1.1036	0.04415		
*Total*	32	10.7034			

**Table 4:** Analysis of variance linear model for R_a_ against processing parameters.

Source	*df*	Sum of Squares	Mean Squares	F-Value	*p*-Value
*Model*	4	7,334,374	1,833,593	2.01	0.12
*Linear*	3	4,691,865	1,563,955	1.72	0.186
Mould Temperature	1	4,277,081	4,277,081	4.7	0.039
Injection Speed	1	404,925	404,925	0.44	0.51
Back-pressure	1	9858	9858	0.01	0.918
*2-Way Interactions*	1	2,642,509	2,642,509	2.9	0.1
Injection Speed × Back pressure	1	2,642,509	2,642,509	2.9	0.1
*Residual*	28	25,507,360	910,977		
*Curvature*	1	0	0	0	1
*Lack-of-Fit*	27	25,507,359	944,717		
*Total*	32	32,841,733			

**Table 5:** Analysis of variance linear model for R_q_ against processing parameters.

Source	*df*	Sum of Squares	Mean Squares	F-Value	*p*-Value
*Model*	4	12,792,135	3,198,034	2.31	0.082
*Linear*	3	8,473,030	2,824,343	2.04	0.13
Mould Temperature	1	7,381,443	7,381,443	5.34	0.028
Injection Speed	1	1,046,061	1,046,061	0.76	0.392
Back Pressure	1	45,527	45,527	0.03	0.857
*2-Way Interactions*	1	4,319,105	4,319,105	3.13	0.088
Injection Speed × Back Pressure	1	4,319,105	4,319,105	3.13	0.088
*Residual*	28	38,689,194	1,381,757		
*Curvature*	1	1523	1523	0	0.974
*Lack-of-Fit*	27	38,687,671	1,432,877		
*Total*	32	51,481,329			

## Appendix A

Table A1 is the DoE used in this research. It has 5 input processing parameters; with a low and high settings for each along with randomised run order to ensure repeatability (refer to Table 1). Table A2 shows the regression models for each of the 4 output parameters, using the 5 main input processing parameters; derived from the DoE data used in this research.

**Table A1:** 2^5^ DoE for the polymer used in this research.

Experimental Number	Run Order	Mould Temperatures (°C)	Injection Speeds (mm/s)	Back-Pressure (MPa)	Melt Temperature (°C)	Holding Time (s)
30	1	90.0	100.0	16.0	260.0	7.0
32	2	90.0	267.8	16.0	260.0	7.0
18	3	90.0	100.0	12.0	155.0	7.0
10	4	90.0	100.0	12.0	260.0	0.0
22	5	90.0	100.0	16.0	155.0	7.0
2	6	90.0	100.0	12.0	155.0	0.0
13	7	25.0	100.0	16.0	260.0	0.0
14	8	90.0	100.0	16.0	260.0	0.0
24	9	90.0	267.8	16.0	155.0	7.0
28	10	90.0	267.8	12.0	260.0	7.0
11	11	25.0	267.8	12.0	260.0	0.0
4	12	90.0	267.8	12.0	155.0	0.0
12	13	90.0	267.8	12.0	260.0	0.0
8	14	90.0	267.8	16.0	155.0	0.0
27	15	25.0	267.8	12.0	260.0	7.0
15	16	25.0	267.8	16.0	260.0	0.0
20	17	90.0	267.8	12.0	155.0	7.0
29	18	25.0	100.0	16.0	260.0	7.0
26	19	90.0	100.0	12.0	260.0	7.0
33	20	57.5	183.9	14.0	207.5	3.5
31	21	25.0	267.8	16.0	260.0	7.0
17	22	25.0	100.0	12.0	155.0	7.0
1	23	25.0	100.0	12.0	155.0	0.0
25	24	25.0	100.0	12.0	260.0	7.0
23	25	25.0	267.8	16.0	155.0	7.0
3	26	25.0	267.8	12.0	155.0	0.0
6	27	90.0	100.0	16.0	155.0	0.0
16	28	90.0	267.8	16.0	260.0	0.0
19	29	25.0	267.8	12.0	155.0	7.0
9	30	25.0	100.0	12.0	260.0	0.0
5	31	25.0	100.0	16.0	155.0	0.0
21	32	25.0	100.0	16.0	155.0	7.0
7	33	25.0	267.8	16.0	155.0	0.0

**Table A2:** Regression models derived from the DoE factorial design analysis.

Response	Regression model (A: Mould Temperature, B: Injection Speed, C: Back-Pressure, D: Melt Temperature and E: Holding Time
Young’s Modulus (**E**)	E = 1434 + 1.837A − 1.383B + 29.9C + 0.661D − 31.87E + 0.00450B × D + 0.0644B × E − 0.1464C × D + 0.1234D × E
Ultimate Tensile Strength (**S_u_**)	S_u_ = 17.495 + 0.00056A − 0.001306B − 0.0170C − 0.01109D − 0.0977E + 0.000825D × E
Arithmetic Mean Deviation (**R_a_**)	R_a_ = −3747 + 11.25A + 22.6B + 324C − 1.71B × C
Root Mean Deviation (**R_q_**)	R_q_ = −4826 + 14.78A + 28.5B + 421C − 2.19B × C
